# Prolonged Photobiomodulation with Deep Red Light Mitigates Incipient Retinal Deterioration in a Mouse Model of Type 2 Diabetes

**DOI:** 10.3390/ijms252212128

**Published:** 2024-11-12

**Authors:** Gabriela Opazo, Felipe Tapia, Alejandra Díaz, Alex H. Vielma, Oliver Schmachtenberg

**Affiliations:** 1 Centro Interdisciplinario de Neurociencia de Valparaíso, Universidad de Valparaíso, Valparaíso 2360102, Chile; 2 Instituto de Biología, Facultad de Ciencias, Universidad de Valparaíso, Valparaíso 2360102, Chile

**Keywords:** diabetes, retina, photobiomodulation, diabetic retinopathy, electroretinogram, gliosis, retinal dysfunction, neurodegeneration, db/db mouse

## Abstract

Diabetic retinopathy is a prevalent complication of type 2 diabetes mellitus, characterized by progressive damage to the retinal structure and function. Photobiomodulation therapy, using red or near-infrared light, has been proposed as a non-invasive intervention to mitigate retinal damage, but has been tested generally with short-term stimuli. This study aimed to evaluate the effects of prolonged photobiomodulation with deep red light on retinal structure and function in a type 2 diabetes mouse model. Transgenic LepRdb/J (db/db) mice were exposed to photobiomodulation therapy via LED devices emitting 654 nm light for 12 h daily over ten weeks and compared to untreated mice. Retinal function was evaluated by flash electroretinography, while structural changes were assessed through histology and immunohistochemistry to detect astro- and microgliosis. At 33 weeks of age, db/db mice were obese and severely diabetic, but exhibited only incipient indicators of retinal deterioration. Electroretinogram b-wave peak latencies were prolonged at intermediate flash intensities, while the outer plexiform layer displayed significantly elevated IBA1 expression. Photobiomodulation therapy prevented these two markers of early retinal deterioration but had no effect on other morphological and functional parameters. Photobiomodulation is well-tolerated and maintains potential as a complementary treatment option for diabetic retinopathy but requires further optimization of therapeutic settings and combinatory treatment approaches.

## 1. Introduction

Diabetic retinopathy (DR) is one of the leading causes of vision impairment and blindness worldwide, affecting individuals with type 1 and type 2 diabetes [1]. It is characterized by progressive alterations in retinal vasculature, neuronal degeneration, and glial activation, ultimately leading to significant retinal dysfunction [2]. The pathophysiology of DR involves a complex interplay of hyperglycemia-induced oxidative stress, inflammation, and neurovascular degeneration, which cumulatively disrupt the structural and functional integrity of the retina [3,4,5,6]. Despite significant advances in managing DR, effective therapeutic strategies to halt or reverse DR progression remain invasive and limited, underscoring the need for novel interventions that can preserve and restore retinal function [7].

Photobiomodulation (PBM) therapy, which generally involves the use of low-intensity infrared, far-red or deep red light, has gained attention as a potential neuroprotective treatment in various models of retinal and neural degenerative diseases [8,9]. PBM is hypothesized to exert its effects by enhancing mitochondrial function, promoting cellular energy production, modulating oxidative stress and reducing cellular inflammation [10,11]. In retinal tissues, these mechanisms may contribute to preserving photoreceptor function and maintaining retinal cell viability under pathological conditions. However, preclinical and clinical studies have provided mixed results on the efficacy of PBM to mitigate retinal damage in pathologies such as age-related macular degeneration, optic neuropathy and myopia [12,13,14,15]. Accordingly, its effectiveness in the context of DR, where both neuroinflammatory and microvascular factors are involved, has been the subject of several studies with overall promising results, but remains to be fully established [16,17,18,19].

The mechanisms underlying the impact of PBM on retinal function are complex and may involve stimulation of cellular energy production, modulation of neuroinflammatory pathways, and the reduction of apoptotic signaling. A significant number of studies have employed a variety of stimulus protocols, intensities, wavelengths, and treatment durations, with repeated short-term PBM applications being the most common approach [20,21]. Previous studies on the cellular targets of PBM in the retina have examined photoreceptor survival and glial cell modulation, with variable but again mostly positive results depending on the model system and light parameters used, such as wavelength, irradiance, and treatment duration [22,23,24].

Nonetheless, significant gaps in knowledge remain regarding the application of PBM in DR, particularly in understanding its effects on retinal functional parameters and its ability to modulate neuroinflammatory markers such as glial fibrillary acidic protein (GFAP) and ionized calcium-binding adapter molecule 1 (IBA1), which are indicative of glial and microglial activity, respectively. Additionally, whether PBM can influence the structural integrity of retinal layers in the context of DR is not well understood. Data on the impact of PBM on retinal electrophysiology under diabetic conditions remain particularly sparse [25,26]. Electroretinography (ERG) serves as a critical tool in assessing retinal signal processing, providing insights into the electrical activity of photoreceptors and inner retinal cells in response to light stimuli. Changes in ERG parameters, such as a- and b-wave amplitudes and peak latencies (implicit times), are indicative of alterations in retinal function and are commonly used to assess the impact of neuroprotective therapies in retinal disease models [27].

This study aimed to address these gaps by evaluating the effects of PBM therapy with continuous daily stimulation on retinal structure and function in a transgenic mouse model of obesity and type 2 diabetes, which has been reported to reproduce features of the neurodegenerative processes in DR [28]. Specifically, we sought to determine whether PBM can mitigate the electrophysiological deficits observed in DR as assessed by ERG according to previously established protocols [29] and to explore its impact on glial reactivity and retinal layer thickness. By examining these parameters, this study seeks to provide a better understanding of the therapeutic potential of PBM in DR and to identify possible avenues for optimizing this intervention to preserve retinal health in diabetic patients.

## 2. Results

### 2.1. Effects of Aging and Diabetes on ERG Parameters, Retinal Morphology and Gliosis Indicators

**Figure 1:**
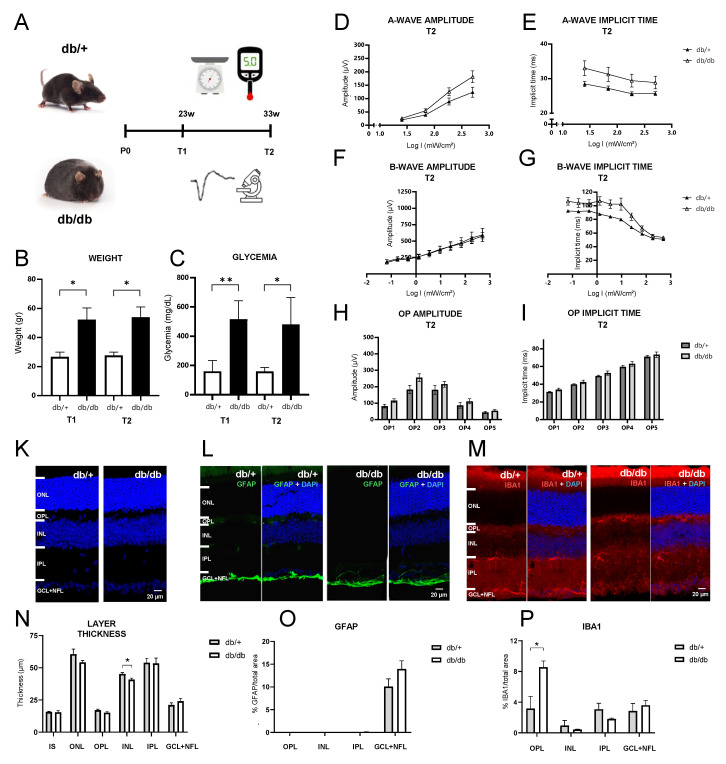
Experimental strategy, evaluation of diabetic status and analysis of retinal characteristics of db/db mice at 33 weeks of age. (**A**) db/+ and diabetic db/db mice were weighed and their fasting glycemia was measured at the start (T1 = 23 weeks of age) and the end (T2 = 33 weeks of age) of the PBM procedure. (**B**,**C**) At T1 and T2, weight and fasting blood glucose levels were significantly higher in db/db mice, confirming obesity and diabetes. (**D**–**I**) At T2, a-wave, b-wave and oscillatory potential (OP) amplitudes displayed no statistical differences between the two groups. Neither did a-wave nor OP implicit times. However, b-wave implicit times (**G**) were significantly elevated at intermediate flash intensities in diabetic mice. (**K**,**N**) Gross retinal morphology was similar between db/+ and db/db mice at T2. Only the INL was slightly thinner in db/db mice. (**L**,**O**) GFAP immunohistochemistry labeled only the GCL+NFL layers, without a significant difference between the groups. (**M**,**P**) IBA1 immunohistochemistry revealed significantly stronger labeling in the OPL of diabetic mice, while the inner retinal layers displayed no significant difference. (* *p* < 0.05, ** *p* < 0.01).

Db/db mice exhibited higher weight and marked hyperglycemia compared to db/+ controls, confirming the development of diabetes in db/db mice (Figure 1B,C). ERG parameters including a-wave amplitude and implicit time (peak latency), b-wave amplitude and implicit time, and oscillatory potential (OP) amplitude and implicit time were then assessed to evaluate functional alterations due to DR (Figure 1D–I). No significant differences were observed between the db/+ and db/db groups regarding a-wave amplitudes (F(1,12) = 3.25, *p* = 0.08, repeated measures ANOVA) or implicit times (F(1,12) = 2.75, *p* = 0.12, repeated measures ANOVA) (Figure 1D,E). b-wave amplitudes displayed no significant differences between db/+ and db/db mice either (F(1,12) = 0.02, *p* = 0.9, repeated measures ANOVA) (Figure 1F). Importantly, while the overall difference in b-wave implicit times between db/+ and db/db mice was just above the significance threshold (F(1,12) = 4.55, *p* = 0.05, repeated measures ANOVA), the interaction term evidenced a decrease in b-wave implicit times across intermediate flash intensities compared to non-diabetic mice (F(9,108) = 4.63, *p* = 0.03, repeated measures ANOVA) (Figure 1G), which reflects a faster ON-bipolar cell response to the flash stimuli. For the OPs, indicators of amacrine-to-bipolar-cell feedback signaling, neither amplitude nor implicit times showed significant differences between the two genotypes in pairwise comparisons (Figure 1H,I).

Gross retinal morphology appeared unaltered in the diabetic mice, and quantitative analysis of the retinal layer thickness revealed only a minor reduction of INL thickness (t(5) = −2.64, *p* = 0.046, *t*-test) (Figure 1K,N). GFAP labeling was absent from retinal Müller cells and only observed in the ganglion and nerve fiber layer in both groups, without a significant difference (Figure 1L,O). However, IBA1 labeling, an indicator of microgliosis, showed significantly elevated expression in the OPL in diabetic mice compared to controls (t(5) = 2.72, *p* = 0.04, *t*-test), while the inner retinal layers displayed some labeling, but no difference in labeled area (Figure 1M,P).

Overall, these findings indicate that diabetes had only minor effects on ERG parameters, retinal morphology and gliosis indicators in db/db mice at 33 weeks of age in our study. Nonetheless, significant differences between db/+ and db/db mice regarding b-wave implicit time at intermediate intensities, and in the expression of IBA1 in the OPL, are indicative of incipient pathology and suggest an early deterioration of photoreceptor to bipolar cell signaling.

### 2.2. Effects of PBM in Diabetic Mice Measured by ERG

**Figure 2:**
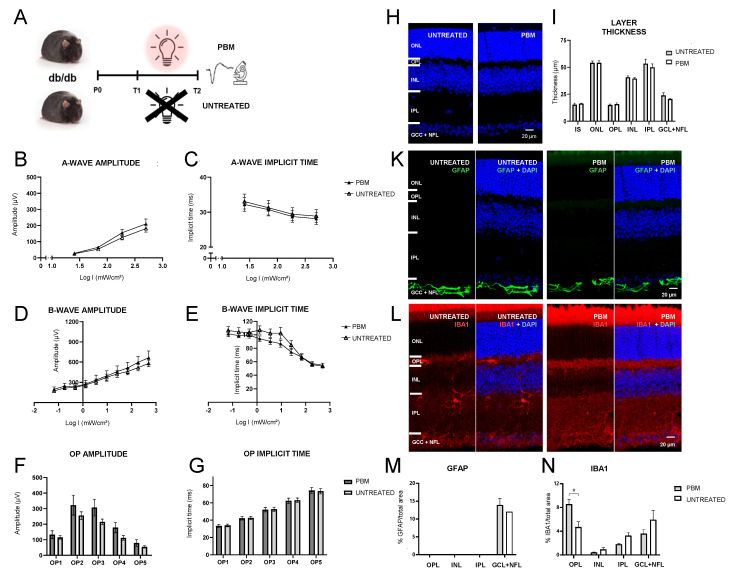
(**A**) Comparison of key ERG, histological, and immunohistological parameters in untreated and PBM-treated db/db mice. (**B**–**E**) No significant differences were observed regarding a-wave amplitude, a-wave implicit time, or b-wave amplitude. However, b-wave implicit times were significantly shorter in the PBM group for four intermediate flash intensities. (**F**,**G**) OP amplitude and implicit time were also unaffected by PBM. (**H**,**I**) Analysis of gross retinal morphology showed no significant effect on retinal layer thickness. (**K**,**L**) GFAP and IBA1 immunolabeling of retinas from untreated and PBM-treated diabetic mice showed no evident qualitative difference. (**M**) Quantitative analysis revealed no difference in GFAP labeling. (**N**) IBA1 labeling was significantly reduced in the OPL of PBM treated diabetic mice, while inner retinal layers displayed no difference. (* *p* < 0.05).

After assessing functional changes associated with DR in db/db mice, the impact of PBM therapy on ERG parameters, retinal morphology and gliosis indicators was evaluated (Figure 2A). No significant differences in a-wave amplitudes (F(1,12) = 0.83, *p* = 0.38, repeated measures ANOVA), implicit times (F(1,12) = 0.06, *p* = 0.8, repeated measures ANOVA), or b-wave amplitudes (F(1,12) = 0.25, *p* = 0.63, repeated measures ANOVA) were observed between the untreated and the PBM-treated group (Figure 2B–D). However, a shorter b-wave implicit time was observed at intermediate flash intensities in the PBM group as evidenced by a significant interaction term (F(9,108) = 3.7, *p* = 0.04, repeated measures ANOVA), even though an overall group difference was not observed (F(1,12) = 0.83, *p* = 0.38, repeated measures ANOVA) (Figure 2E). OP amplitudes and implicit times were unaffected by PBM (Figure 2F,G).

In the histological analysis, PBM showed no effect on gross retinal morphology and individual layer thickness (Figure 2H,I). Furthermore, GFAP remained unaltered in the GCL+NFL and absent from other retinal layers (Figure 2K,M). However, IBA1 labeling displayed significantly reduced expression in the OPL compared to untreated mice (t(6) = 2.91, *p* = 0.03, *t*-test), at levels similar to non-diabetic control mice (Figure 2L,N). These findings indicate that PBM therapy mitigated the incipient retinal deterioration evidenced by prolonged b-wave peak latencies and prevented the development of microgliosis, as evidenced by significantly reduced IBA1 labeling.

## 3. Discussion

DR is a complex retinal disorder that arises from chronic hyperglycemia, leading to progressive damage to retinal cells and microvasculature. The early stages of DR are characterized by subtle alterations in retinal signaling and the activation of glial cells, which contribute to inflammation and neurodegeneration [2]. As DR progresses, these changes lead to significant functional impairment and structural damage, ultimately causing functional blindness. Current therapeutic options are limited, invasive, or focus primarily on managing later stages of the disease, underscoring the need for novel interventions that target early retinal dysfunction [30].

This study was designed to examine the effects of continuous daily PBM therapy using deep red light, as opposed to the application of repeated short PBM stimuli, on retinal structure and function in a type 2 diabetic mouse model. Our findings indicate that while PBM therapy provided some protective effects against retinal deterioration in diabetic mice, its overall impact on functional and structural markers of DR was modest. We first sought to confirm evidence for DR progression in our obesity and type 2 diabetes mouse model. db/db mice exhibited mild signs of retinal deterioration at 33 weeks of age in the present study. This finding is consistent with previous studies suggesting that the onset of retinal degeneration in this model is gradual and occurs over an extended period [31], but partly at odds with a prior report characterizing the retina of the db/db mouse, which observed more significant retinal changes at an earlier stage [28]. In particular, our ERG results showed that b-wave peak latencies were prolonged at intermediate flash intensities in diabetic mice compared to non-diabetic controls, indicating initial disruptions in photoreceptor-to-bipolar cell signaling, resulting in a slower ON bipolar cell response. Additionally, immunohistochemical analysis revealed increased expression of IBA1 in the outer plexiform layer of db/db mice, suggesting enhanced microglial activation, a hallmark of retinal inflammation in DR. These results are consistent with the literature, which highlights the role of neuroinflammation and microglial activation in the pathogenesis of DR [32,33].

The primary novel finding of this study is the ability of PBM therapy to mitigate these early signs of retinal dysfunction. PBM-treated mice displayed significantly lower b-wave latencies at intermediate flash intensities compared to untreated db/db mice, suggesting a protective effect on retinal signaling pathways. Moreover, PBM therapy significantly reduced IBA1 expression in the OPL of diabetic mice, indicating that PBM may have anti-inflammatory effects in the diabetic retina. These results align with previous studies suggesting that PBM can reduce neuroinflammation and promote cellular homeostasis through its effects on mitochondrial function and oxidative stress reduction [10,11].

The observed protective effects of PBM on b-wave latencies and microglial activation in diabetic mice likely stem from its ability to modulate mitochondrial activity and reduce oxidative stress [34]. Mitochondria play a crucial role in cellular energy production, and mitochondrial dysfunction is a key driver of oxidative stress in DR. PBM is generally thought to enhance mitochondrial function by stimulating cytochrome c oxidase, leading to increased ATP production and the reduction of reactive oxygen species (ROS) [11]. By improving mitochondrial efficiency, PBM may reduce oxidative damage to retinal cells, thereby preserving photoreceptor function and reducing inflammation.

In addition to its effects on the mitochondria, PBM may also exert anti-inflammatory effects by modulating neuroinflammatory pathways [35,36,37]. The reduction in IBA1 expression observed in our study suggests that PBM therapy may reduce microglial activation in the diabetic retina. Microglia, the resident immune cells of the central nervous system, are activated in response to diabetic stress and contribute to retinal inflammation through the release of pro-inflammatory cytokines and ROS. PBM has been shown to reduce the production of pro-inflammatory cytokines and increase the expression of neuroprotective factors such as brain-derived neurotrophic factor (BDNF) and glial cell line-derived neurotrophic factor (GDNF) [8]. These effects may help to attenuate the chronic inflammatory response observed in DR, thereby reducing retinal damage.

Altogether, our data suggest that continuous daily PBM application may provide some neuroprotective effects in the early stages of DR, although it may not be sufficient to fully prevent the progression of retinal degeneration. Further optimization of PBM parameters, such as wavelength, irradiance, and treatment duration, may be necessary to enhance its therapeutic efficacy [38]. A few limitations of the study should be considered when interpreting the results. First, the db/db mouse model of type 2 diabetes used in this study displays only mild signs of DR at 33 weeks of age. While this allowed us to investigate the early effects of PBM therapy, it also limits the generalizability of our findings to more advanced stages of the disease. Furthermore, the PBM stimulation of freely moving mice, while avoiding confounding factors due to restraint stress, made it impossible to exactly define the doses received by each animal. Finally, the sample size for histological analyses was relatively small, which may have limited the statistical power to detect subtle changes in retinal structure and gliosis.

The results of this study suggest that PBM therapy holds potential as a non-invasive intervention for mitigating early signs of retinal deterioration in DR. However, further research is needed to optimize PBM parameters [21] and explore its effects in more advanced stages of the disease. Future studies should investigate the mechanisms underlying the neuroprotective effects of PBM in greater detail, particularly its impact on mitochondrial dynamics, neuroinflammatory pathways, and apoptotic signaling. Additionally, combining PBM with other therapeutic strategies, such as anti-inflammatory or anti-VEGF treatments, may enhance its efficacy and provide a more comprehensive approach to preserving retinal health in diabetes.

## 4. Materials and Methods

### 4.1. Animals

Transgenic mice of the BKS.Cg-Dock7m+/+ LepRdb/J line, strain #000642, were acquired from The Jackson Laboratory (Bar Harbor, ME, USA) and used in this study. After breeding, this rodent line produces three phenotypes: homozygous for LepRdb (+ db/+ db), heterozygous for LepRdb (m +/+ db) and misty (m +/m +). Genotypes were determined based on phenotypes and the glucose status. Mice that are homozygous for the mutant Leptin receptor gene and wild type for Dock7 develop obesity and sustained hyperglycemia, making them an ideal model for studying type 2 diabetes mellitus. In contrast, mice heterozygous for LepR and Dock7 develop normally and were used as non-diabetic controls rather than the misty phenotype, which often presents neurological symptoms in addition to its grey coat. Throughout this report, the obese mice were referred to as db/db, and the control non-diabetic mice as db/+. The mice were bred and housed under a 12 h light/dark cycle (about 12 lux during daytime) with unrestricted access to food and water, maintained at a room temperature of 22 °C and 40–60% humidity.

PBM treatment was applied starting at 23 weeks of age and maintained for 10 weeks (Figure 1A and Figure 2A). To verify phenotypic expression differences among the groups, glycemia was evaluated using a commercial glucose analyzer (Accu-Chek, Roche Diagnostics, Indianapolis, IN, USA). Measurements were taken at the end of the ERG session by cutting approximately 1 mm from the tail of the mouse while it was anesthetized with isoflurane according to the ERG protocol. Following this procedure, the weights of the animals were also recorded.

For histological analysis, mice were sacrificed through deep anesthetic induction in a sealed plastic chamber with 5% isoflurane. Reflex loss was monitored by checking for the absence of blink, corneal, and withdrawal reflexes. Euthanasia was immediately performed by decapitation using a guillotine. The experimental protocols and the use of animals in this study were approved by the Bioethics Committee of the University of Valparaíso under the bioethics validation certificate CBC 722022 (2023).

### 4.2. Photobiomodulation

A PBM system was constructed to provide consistent deep red light exposure for the mice. LED devices (Luxeon C, L1C1-DRD1) with a peak wavelength of 654 nm, measured with a spectrometer (USB650, Ocean Insight, Orlando, FL, USA) were positioned above each mouse cage, delivering an average continuous irradiance of 218 μW/cm^2^, measured at the mouse eye level. The PBM system was programmed to deliver light for 12 h daily, coinciding with the light phase of the mouse circadian rhythm, and stimulation was maintained for 70 consecutive days. The stimulation cages were covered with black hoods, and the residual white background light was below 1 lux. The setup was designed to avoid any additional handling of the mice during light exposure in order to prevent potentially confounding stress-induced side effects. Routine maintenance of the cages, including bedding changes and the provision of food and water, was conducted without disrupting the established PBM routine.

### 4.3. Electroretinography (ERG)

Flash ERG was conducted to assess retinal function in both db/+ and db/db mice before and after PBM treatment. Prior to the ERG recordings, mice were dark-adapted for a minimum of 8 h. The ERGs were recorded on the final day of the 70-day PBM period. Mice were anesthetized with 3% isoflurane for induction in a sealed plastic chamber for approximately 3 min and anesthesia was maintained at a concentration of 2% and 2.5% during recordings for the db/+ and db/db mice, respectively, due to significant weight differences between the two genotypes. After anesthesia was confirmed by the absence of withdrawal, blink, and corneal reflexes, 0.5% proparacaine hydrochloride was applied as a topical anesthetic to the corneas, and the pupils were dilated using 1% atropine. To keep the corneas hydrated during the ERG recording, artificial tears (Lacryvisc 3 mg/g, Ophthalmic Gel, Alcon, Fort Worth, TX, USA) were applied immediately before starting the procedure. A custom Ganzfeld dome system was constructed using white LEDs arranged equidistantly around an acrylic dome. The light intensity and duration were controlled via a digitizing board connected to WinWCP version 4.9.4 software (University of Strathclyde, Glasgow, Scotland, UK). Custom-designed electrodes were used for the ERG recording, including a corneal recording electrode made of silver wire, a subdermal reference electrode made of stainless steel placed on the cheek, and a ground electrode made of copper wire connected to the Faraday cage. Mice were stimulated with a series of 10 flashes of 5 ms duration under scotopic conditions, with intensities ranging from −1.1 to +2.69 log mW/cm^2^ (−3.46 to −0.34 log cd×s/m^2^). Each recording included a 200 ms baseline before flash onset. The average of these recordings was reported for each intensity. Although responses to 10 flash intensities were explored, only 4 intensities were reported for the a-wave due to insufficient signal-to-noise ratios for the dimmest flashes. Oscillatory potentials (OPs) were extracted offline from averaged traces obtained at the maximum intensity flash stimulus. The recordings were obtained as described previously [29], using a differential amplifier (Model 3000, AM Systems Inc., Sequim, WA, USA) set to AC mode with a gain of 1000× and band-pass filtered between 1 and 1000 Hz. The mice were positioned on a platform with a thermoregulated base set at 32 °C, and anesthesia was maintained throughout the procedure with an isoflurane nozzle system. Each ERG session lasted approximately 45 min, including time for sedation and execution of the protocol. Post experiment, the weight and glycemia of the mouse were measured according to the previously described protocol. Initial data analysis was performed using WinWCP software, and a custom script written in Python 3.11.5 was developed to extract features of the a- and b-waves using a 5th-order Butterworth band-pass filter to isolate specific wave components. A total of 6 db/+ and 14 db/db mice were recorded.

### 4.4. Retinal Immunohistochemistry

To evaluate retinal structure and glial activity, eyes were extracted, and anterior chambers were removed. The eyes were fixed in 4% paraformaldehyde for one hour, washed in PBS buffer (pH 7.4) and cryopreserved in a gradient of 10%, 20%, and 30% sucrose consecutively for 10 min, 20 min, and overnight at 4 °C. Samples were embedded in tissue freezing medium (Tissue-Tek, Sakura Finetek, Tokyo, Japan), and 20 μm transverse sections were prepared using a cryostat (Leica CM-1900, Wetzlar, Germany). Sections were mounted on slides pre-treated with poly-L-lysine to ensure adhesion. The retinal sections were rehydrated in PBS and blocked with a solution containing 1% BSA, 1% horse serum, and 0.3% Triton X-100 in PBS. The sections were incubated with primary antibodies overnight at 4 °C in a humidified chamber. GFAP was labeled using an anti-glial fibrillary acidic protein antibody at a dilution of 1:500 (Cat# 13-0300, Thermo Fisher, Waltham, MA, USA), and microglia were labeled with an anti-IBA1 antibody at 1:1000 (A104332, Antibodies, Cambridge, UK). After primary antibody incubation, sections were washed in PBS and incubated with secondary antibodies (Alexa fluor 555 anti-rabbit for IBA1 (AB150078, Waltham, MA, USA, Abcam) and Alexa fluor 488 anti-rat for GFAP (AB150157, Abcam)) at room temperature for 1 h, followed by staining with DAPI (0.25 μg/mL) for 10 min. Sections were mounted using a fluorescence mounting medium (Fluoromount, Sigma-Aldrich, St. Louis, MO, USA) and visualized using a Nikon C1 Plus confocal microscope (Nikon C1 Plus, Tokyo, Japan). For quantitative analysis, data were averaged from at least three images per mouse. GFAP and IBA1 activity were approximated by the number of pixels above a subjectively defined threshold relative to the total number of pixels in the delineated retinal area, specifically the ganglion cell layer + nerve fiber layer (GCL+NFL), inner plexiform layer (IPL), inner nuclear layer (INL), and outer plexiform layer (OPL). The thickness of the retinal layers was also measured, with boundaries manually delineated and averaged from at least 20 measurements per section. Images showing severe damage or an altered structure in the layers of interest were excluded from analysis. A total of 4 db/+ and 8 db/db mice were used for histological analysis.

### 4.5. Statistical Analysis

Statistical analysis was performed using Jamovi 2.6.2. For pairwise comparisons, student’s *t*-tests or Mann–Whitney U tests were used depending on the normality of the data assessed using the Shapiro–Wilk test. Weight, glycemia, OPs measurements and histological data were analyzed in this way. On the other hand, serial ERG measurements were analyzed using two-way repeated measures ANOVA, with Greenhouse–Geisser correction for sphericity deviations and Tukey’s post hoc tests. Statistical significance was defined as *p* < 0.05. All graphs were generated using GraphPad Prism 8.0. Error bars in the graphs represent the standard error unless otherwise stated, and significance levels are indicated with asterisks (* *p* < 0.05, ** *p* < 0.01).

## 5. Conclusions

This study evaluated the effects of prolonged daily PBM therapy using deep red light on retinal function and structure in a type 2 diabetic mouse model. Our findings suggest that while db/db mice exhibited only mild signs of retinal dysfunction at 33 weeks, PBM therapy provided protective effects, particularly in reducing b-wave peak latencies and microglial activation in the outer plexiform layer. These results indicate that PBM can mitigate some early indicators of DR, but its overall impact on retinal function and structure appears limited. Thus, while PBM shows promise as a non-invasive intervention for DR, further studies are needed to optimize treatment parameters and assess its efficacy in more advanced disease stages. The protective effects of PBM therapy observed in this study underscore its potential as a complementary treatment for DR. By modulating mitochondrial activity and reducing oxidative stress and inflammation, PBM may preserve retinal function in the early stages of DR. However, given the modest overall impact, its application as a standalone therapy is limited, and the need for combinatory approaches or optimized protocols remains crucial for achieving significant therapeutic benefits in DR management.

Future research should focus on refining PBM protocols, including adjustments to wavelength, irradiance, and treatment duration, to enhance its neuroprotective effects. Studies should also investigate the combination of PBM with other therapeutic strategies, such as anti-inflammatory or anti-VEGF treatments, to provide a more comprehensive approach to treating DR. Additionally, investigating PBM in more advanced stages of DR, along with exploring its underlying mechanisms, may help identify new therapeutic targets and optimize interventions for preserving retinal health in diabetic patients.

## Data Availability

The original datasets used in the current study are available from the corresponding author on reasonable request.
